# An Endoscopic-Assisted Open Removal of a Retained Foreign Body From Frontal Sinus

**DOI:** 10.7759/cureus.25359

**Published:** 2022-05-26

**Authors:** Eirini Nikolaidou, Eleni Karagergou, Spyridon Gougousis, Sophia Papadopoulou, Ioannis Tilaveridis

**Affiliations:** 1 Department of Plastic, Reconstructive and Hand Surgery & Burns ICU, General Hospital of Thessaloniki “G. Papanikolaou”, Thessaloniki, GRC; 2 Department of Otorhinolaryngology - Head and Neck Surgery, General Hospital of Thessaloniki “G. Papanikolaou”, Thessaloniki, GRC; 3 Department of Plastic, Reconstructive and Hand Surgery & Burns ICU, General Hospital of Thessaloniki "G. Papanikolaou", Thessaloniki, GRC; 4 Department of Oral and Maxillofacial Surgery, Aristotle University of Thessaloniki, Thessaloniki, GRC

**Keywords:** open surgical approach, transnasal endoscopy, penetrating injury, foreign body, frontal sinus

## Abstract

Facial penetrating injuries can cause retention of foreign bodies in the frontal sinus. This rare condition can remain underdiagnosed for years, since non-specific symptoms, such as headaches and nasal obstruction, can be developed. So far, removal by an endoscopic approach is the most preferred treatment option because it is less invasive with a short recovery time. However, removal by an open surgical approach remains the method of choice for large foreign bodies, especially in cases of coexistent non-reducible fractures of the anterior table of the frontal bone. We present a case where a combined approach - open and endoscopic - was necessary to successfully remove a retained foreign body from a frontal sinus. With the assistance of a transnasal endoscope, the retained stone was mobilized and removed from the open frontal sinus followed by osteosynthesis of the anterior table. Therefore, in special circumstances, a combination of both techniques should be considered for the optimal outcome.

## Introduction

The frontal sinus is frequently involved in facial trauma due to its prominent position on the face [1]. However, penetrating injuries causing retention of foreign bodies in this anatomical region are rare and they usually involve retained gunshot pellets, stones, and glass particles [2-6]. The presence of a foreign body in the frontal sinus can be asymptomatic or accompanied by non-specific symptoms, such as headaches, nasal obstruction, and intermittent nasal hemorrhage [7]. Therefore, these injuries can remain underdiagnosed for years [7]. So far, removal by a transnasal endoscopic approach is the most preferred treatment option [8,9]. However, this approach might not be efficient in cases of large retained objects and an open technique through the anterior table of the frontal sinus might be necessary. In this case report, we present a combined technique - open and endoscopic - which was deemed necessary intraoperatively to successfully remove a retained foreign body from a frontal sinus, which was not possible to remove by an open approach.

## Case presentation

Α 61-year-old male patient was referred to our clinic with a retained foreign body in his right frontal sinus after a fall he sustained during mountain hiking a month ago. At that time, he had presented to the emergency department of a peripheral hospital where the wound was closed without further imaging. A few days later, the patient suffered from a headache over the right eyebrow and right nasal obstruction. He also complained of fluid constantly draining from his healed wound. No other symptoms were noticed.

Physical examination revealed a 6-cm vertical, well-healed scar at the forehead, over his right eyebrow with a palpable step off at the inferior part of it, and a skin disruption that was hardly noticed and which was discharging a small amount of clear fluid. Both vision and extraocular muscle movements were intact.

**Figure 1:**
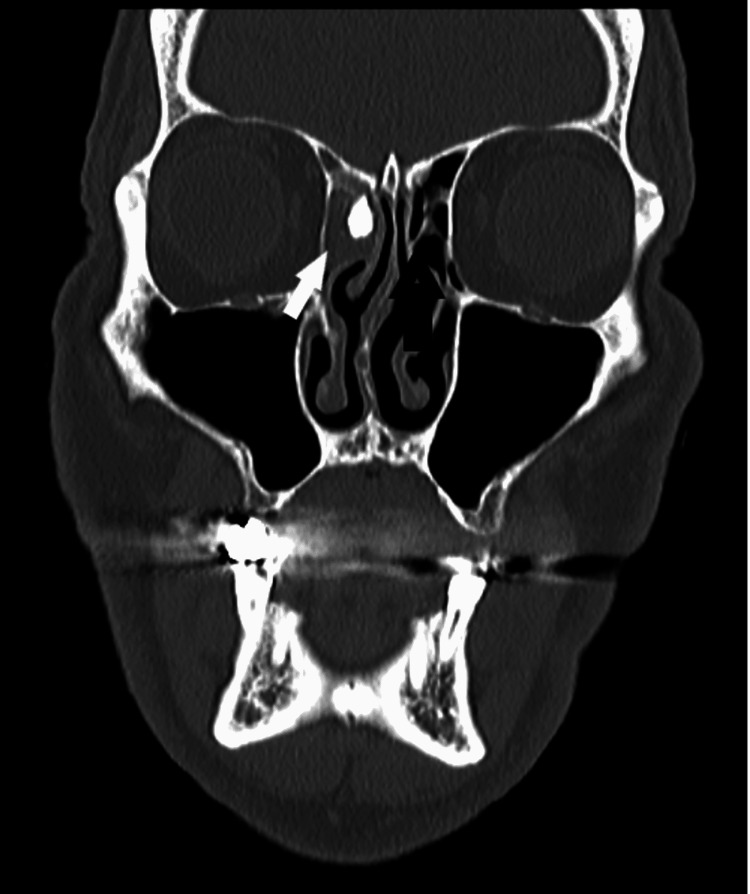
Bone window of the coronal computed tomography (CT) scan revealed a hyperdense calcified structure (arrow), consistent with a retained foreign body at the upper part of the anterior ethmoid cells.

Computed tomography (CT) scan revealed the presence of a comminuted fracture of the anterior table of the right frontal sinus with bone segments within it and the formation of a sinocutaneous fistula. Pneumatization loss and non-homogeneous soft tissue mass signs were noticed in the right frontal sinus. A hyperdense calcified structure, consistent with a retained foreign body, was also noticed at the upper part of the frontal recess (Figure 1). 

**Figure 2:**
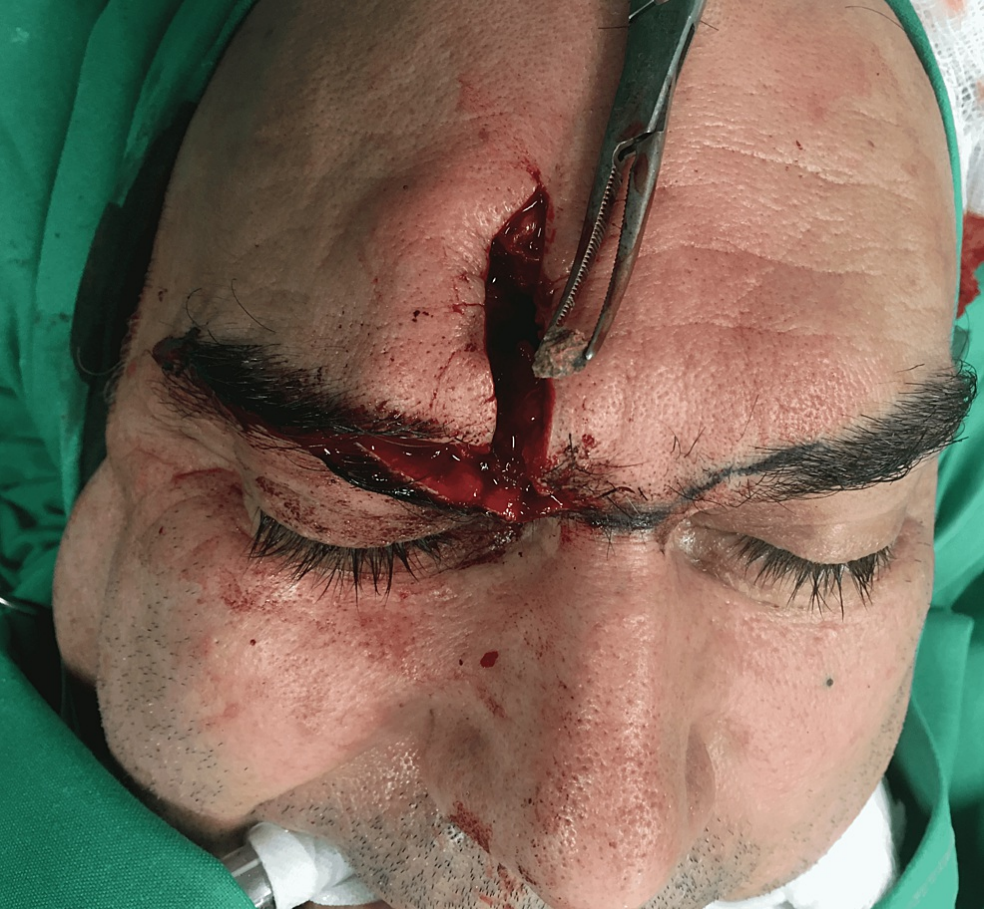
Removal of the foreign body (stone) using an endoscopic-assisted open technique.

Under general anesthesia, through his scar over his right eyebrow, a frontal sinusotomy was performed and the retained foreign body was identified as lodged at the frontal ostrium. Not being able to remove it openly, a transnasal endoscope was used to provide visual access to the frontal recess. First, an endoscopic inspection of the nasal cavity was performed and the frontal recess was reached via the intact bulla technique. This decision was made to avoid injury to the anterior ethmoid artery. The middle turbinate, the uncinate process, and the bulla ethmoidalis were identified. Then an uncinectomy and an antrostomy were performed to identify the superior wall of the maxillary sinus and the lamina papyracea. After the removal of some anterior ethmoid cells, the natural ostium of the frontal sinus was reached. The foreign body (about 1 cm in size) was then visible but wedged in the frontal recess and had to be mobilized with the endoscope, to remove it through the open frontal sinus. Thorough irrigation was followed, osteosynthesis of the anterior table of the sinus was performed, and the old scar was removed (Figure 2).

**Figure 3:**
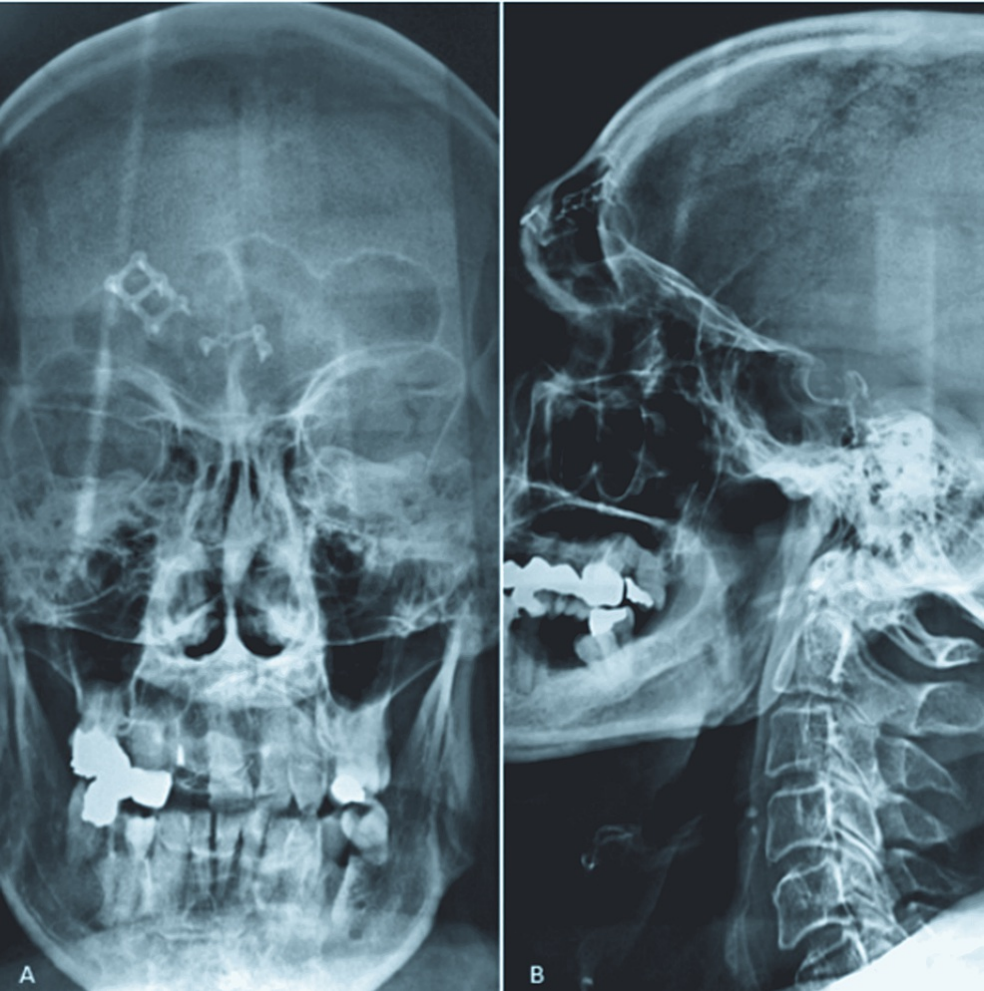
Post-operative x-ray showing the re-pneumatization of the right frontal sinus after six weeks of stenting of the nasofrontal duct (A: en face x-ray; B: profile x-ray).

A plastic tube was placed in the nasofrontal duct to help maintain patency. After six weeks of stenting, the tube was removed and re-pneumatization of the right frontal sinus was confirmed with an x-ray (Figure 3).

At one year of follow-up, the patient’s symptoms disappeared completely and the esthetic outcome was satisfactory.

## Discussion

Foreign bodies in the frontal sinus after skin lacerations and frontal fractures are rarely encountered. When these injuries are not properly explored, retained foreign bodies can lead to sinocutaneous fistulas with non-specific symptoms, such as headaches, nasal obstruction, and intermittent nasal hemorrhage [7]. When complicated, they may also cause improper drainage of the sinuses, secondary infections and sinusitis, or even inflammation of the dura [9]. To avoid those detrimental sequelae, a high index of suspicion is required and early removal is recommended.

In 2012, Yarlagadda et al. proposed a diagnostic algorithm for retrieval of projected foreign bodies from the paranasal sinuses, concluding that pre-operative imaging investigation is mandatory for determining the exact location of the foreign body [10]. Axial CT scanning is the examination of choice since it can precisely localize the foreign body, assess the integrity of the anterior and posterior frontal sinus wall, and diagnose secondary complications, such as intracranial penetration and sinusitis [11]. 

Regarding surgery, the open approach was the first surgical technique described for the removal of a piece of glass in the frontal sinus [5]. A few years later, the advances in endoscopic techniques including transnasal endoscopy resulted in their wide use for the treatment of frontal sinus pathologies, such as chronic rhinosinusitis recalcitrant to medical management or retained foreign bodies in sinuses [12,13]. Nowadays, transnasal endoscopy has become the standard of care in the removal of foreign bodies from sinuses due to its main advantages - enhanced visualization, less invasiveness, short recovery time, and reduced complication rate [13]. Several endoscopic techniques have been described for this reason including irrigation of the frontal sinus with a curved antral cannula to launch and wash out small pieces of glass from the frontal sinus into the middle meatus [11]. The newest surgical techniques use the Draf IIa procedure, which is based on anterior ethmoidectomy and dissection of all anterior fronto-ethmoidal cells within the frontal recess [8]. In our case, the intact bulla technique that was used reduced the possibility of anterior skull base injury, anterior ethmoid artery damage, and papyraceous lamina laceration. 

When the retained foreign body looks fragmented and the nasofrontal ductus is blocked, the endoscopic approach is contraindicated [14]. Also, in cases of retained large objects, an endoscopic removal might not be feasible and an open surgical technique is indicated. In addition, we believe that non-reducible fractures at the anterior table of the frontal bone that need reduction and fixation as well as the presence of unsightly scars or sinocutaneous fistulas that need excision should be considered as relative indications for an open approach.

In the presented case, the lodged position of the retained stone in the frontal recess could not allow an open removal from the frontal sinus and the size of the foreign body could not allow its endoscopic removal. Hence, the endoscope effectively pushed the stone back to the frontal sinus, making its removal easy.

## Conclusions

Despite the fact that foreign bodies in the frontal sinus are rare, facial trauma history and non-specific symptoms raise clinical suspicion of a retained foreign body. Axial CT is the gold standard for the diagnosis of this condition. Although transnasal endoscopy has become the standard of care in the removal of sinuses' foreign bodies, open surgical removal is still occasionally indicated, especially in cases of large foreign bodies (>1 cm) and non-reducible fractures of the frontal bone that need revision. This case report presents a unique combination of both techniques and points out an important technical tip that can be useful in special circumstances. 

## References

[1] Kaplan AS, Green JD Jr, McCaffrey TV (1989). Unsuspected foreign body in the frontal sinus and anterior cranial fossa. Ann Emerg Med.

[2] Payne RF (1967). Foreign bodies in the frontal sinus. Br J Radiol.

[3] Garces SM, Norris CW (1972). Unusual frontal sinus foreign body. J Laryngol Otol.

[4] Gadre KCL, Odaya JD, Deshpande CK (1963). Foreign body (stone) in the frontal sinus. J Laryngol Otol.

[5] Onerci M, Oğretmenoğlu O, Yilmaz T (1997). Glass in the frontal sinus: report of three cases. J Laryngol Otol.

[6] Popli G, Dubey P, Bansal V, Bansal A, Gupta T, Kapoor S (2016). Impacted foreign body in frontal sinus - a rare case report. J Med Sci Clin Res.

[7] Cukurova I, Demirhan E, Gumussoy M, Yalcin Y, Yigitbasi OG (2013). Foreign body in frontal sinus: case report. Turk Arch Otolaryngol.

[8] Levy DA, Lee AY, Abuzeid WM, Akbar NA (2020). Guns n' noses: endoscopic removal of an air-gun pellet retained in the frontal sinus. Ear Nose Throat J.

[9] Nataraj R, Jagade M, Chavan R (2015). An unusual maxillary sinus foreign body and its endoscopic assisted removal. Int J Otolaryngol Head Neck Surg.

[10] Yarlagadda B, Jalisi S, Burke P, Platt M (2012). Retrieval of projectile foreign bodies from the paranasal sinuses and skull base. Am J Rhinol Allergy.

[11] Tosun F, Ozkaptan Y (2000). Practical approach to foreign bodies in the frontal sinus. Otolaryngol Head Neck Surg.

[12] Korban ZR, Casiano RR (2016). Standard endoscopic approaches in frontal sinus surgery: technical pearls and approach selection. Otolaryngol Clin North Am.

[13] Gross M, Regev E, Hamdan K, Eliashar R (2005). Penetrating rubber bullet into the ethmoid sinus: should the bullet be removed?. Otolaryngol Head Neck Surg.

[14] Sedat A, Sanli A, Eken M, Hardal U (2009). Glass particles in the frontal sinus. Turk J Med Sci.

